# Developed Chitosan/Oregano Essential Oil Biocomposite Packaging Film Enhanced by Cellulose Nanofibril

**DOI:** 10.3390/polym12081780

**Published:** 2020-08-09

**Authors:** Shunli Chen, Min Wu, Caixia Wang, Shun Yan, Peng Lu, Shuangfei Wang

**Affiliations:** 1 College of Light Industry and Food Engineering, Guangxi University, Nanning 530004, China; chenshunli@st.gxu.edu.cn (S.C.); wangcx@st.gxu.edu.cn (C.W.); yanshun2020gxu@163.com (S.Y.); lupeng@gxu.edu.cn (P.L.); 2 Guangxi Key Laboratory of Clean Pulp & Papermaking and Pollution Control, Nanning 530004, China

**Keywords:** cellulose nanofibrils, chitosan, oregano essential oil, antimicrobial, oxygen barrier properties

## Abstract

The use of advanced and eco-friendly materials has become a trend in the field of food packaging. Cellulose nanofibrils (CNFs) were prepared from bleached bagasse pulp board by a mechanical grinding method and were used to enhance the properties of a chitosan/oregano essential oil (OEO) biocomposite packaging film. The growth inhibition rate of the developed films with 2% (*w*/*w*) OEO against *E. coli* and *L. monocytogenes* reached 99%. With the increased levels of added CNFs, the fibrous network structure of the films became more obvious, as was determined by SEM and the formation of strong hydrogen bonds between CNFs and chitosan was observed in FTIR spectra, while the XRD pattern suggested that the strength of diffraction peaks and crystallinity of the films slightly increased. The addition of 20% CNFs contributed to an oxygen-transmission rate reduction of 5.96 cc/m^2^·day and water vapor transmission rate reduction of 741.49 g/m^2^·day. However, the increase in CNFs contents did not significantly improve the barrier properties of the film. The addition of 60% CNFs significantly improved the barrier properties of the film to light and exhibited the lowest light transmittance (28.53%) at 600 nm. Addition of CNFs to the chitosan/OEO film significantly improved tensile strength and the addition of 60% CNFs contributed to an increase of 16.80 MPa in tensile strength. The developed chitosan/oregano essential oil/CNFs biocomposite film with favorable properties and antibacterial activity can be used as a green, functional material in the food-packaging field. It has the potential to improve food quality and extend food shelf life.

## 1. Introduction

Petroleum-based plastic films have been widely used in recent decades in the packaging field due to their low cost, good chemical stability and excellent barrier performance [1]. However, the use of non-biodegradable materials in packaging applications has raised concerns about environmental pollution. The demand for advanced and eco-friendly packaging materials owing to their excellent physical, mechanical, and barrier properties and antimicrobial activity is significantly increasing, especially with increased consumer awareness of environmental protection and with increased attention being paid to food quality and safety. Recently, biodegradable materials, such as chitosan [2,3,4], starch [5], pectin [6], gelatine [7] and cellulose [8], as matrices to develop biologic composite packaging materials with good oxygen, water vapor barrier properties, antibacterial properties and mechanical properties, have become a focus of scholars. However, biodegradable packaging films prepared by a single biomass material often cannot simultaneously possess a variety of favorable properties which limits the application of biomass packaging materials in the field of food packaging. Therefore, two or more kinds of material are blended together, and the functional components are added to prepare biocomposite packaging materials with good mechanical properties, oxygen and water vapor barrier properties and antibacterial or antioxidant properties.

Chitosan, which consists of (1,4)-linked-2-amino-deoxy-b-d-glucan, is a kind of cationic polysaccharide and is the deacetylated form of chitin [9]. As a renewable natural biopolymer, chitosan is derived from various sources and exhibits non-toxicity, biocompatibility, biodegradability and excellent film-forming properties. Meanwhile, chitosan has broad-spectrum antimicrobial activity against both Gram-positive and Gram-negative bacteria as well as fungi. Films prepared from chitosan were extensively used in food packaging as a degradable packaging material and inhibited bacterial reproduction, prolonged the shelf life of food and improved food quality and safety [10,11]. However, the poor mechanical and barrier properties of chitosan-based packaging materials compared to those of non-biodegradable materials have limited its widespread usage [12]. In order to develop the application of chitosan-based films in the field of food packaging, physical or chemical modification strategies have been tried. Poverenov et al. [13] prepared the alginate–chitosan coating by layer-by-layer electrostatic deposition, the coating showed excellent gas-exchange and water vapor permeability properties that protected the appearance of the fresh-cut melon. Caseinate and chitosan by ion interaction was carried out to obtain the composite films, which showed improved water vapor barrier properties [14]. The chitosan-grafted salicylic acid films have been found to maintain better quality in cucumber, making it a promising material for food packaging applications [15]. Cellulose nanofibrils (CNFs) from natural resources are recognized as the most abundant, renewable and biodegradable polymeric materials [16] and have been widely used due to their good biocompatibility, chemical stability, mechanical properties and oxygen barrier properties [17,18,19,20]. The addition of 3% (*w*/*w*) of CNFs increased the tensile strength, elongation at breaking and Young’s modulus of corn starch film [21]. The water vapor permeability of bio-nanocomposite films was reduced by incorporating 4% (*w*/*w*) CNFs [22].

The antibacterial properties of chitosan are mainly related to its degree of deacetylation, molecular weight, types of microorganisms and other factors. Sanchez-Gonzalez et al. [23] showed that pure chitosan films had obvious antibacterial effects against *E. coli* and *L. monocytogenes*, but could not inhibit *S. aureus* growth. Song et al. [24] reported that chitosan film exhibited only slight inhibitory activity against *E. coli* and *S. aureus*. Some research showed that the antibacterial properties of chitosan were still controversial and unstable when chitosan was used as a single antibacterial agent when preparing film. Essential oil as a natural antibacterial agent was often added to the film to improve the antibacterial properties of the films. Oregano essential oil (OEO) mainly consists of phenolic compounds [25] that can effectively prevent spoilage and prolong the shelf life of food and is widely used in food packaging due to its favorable antioxidant and antibacterial properties. OEO can be directly incorporated into the matrix of packaging film and is released during transportation and/or storage of food and thereby contributes to reducing food spoilage [26]. However, to our knowledge, no information has been reported regarding the enhancement of cellulose nanofibers on the mechanical, thermal and barrier properties of chitosan/OEO packaging film.

The main objective of this study was to develop chitosan/OEO biocomposite packaging film enhanced by CNFs to obtain favorable physical characteristics and antimicrobial properties. The morphology, chemical structure, physical characteristics and antimicrobial properties of the developed biocomposite packaging films were measured and analyzed. The film will have potential applicability for food packaging and high-value goods.

## 2. Materials and Methods

### 2.1. Chemicals and Materials

Chitosan powder (80%–95%, deacetylated) was purchased from Sinopharm Chemical Reagent Co., Ltd., Shanghai, China. Pure OEO and Tween-80 were obtained from Shanghai Aladdin Bio-Chem Technology Co., Ltd., Shanghai, China. *E. coli* (ATCC 25,922) and *L. monocytogenes* (ATCC 19,115) strains were obtained from the China Center of Industrial Culture Collection, Beijing, China. Bleached bagasse pulp board was purchased from Guangxi GuiTang (Group) Co., Ltd, Guigang, Guangxi, China. All other chemicals were of analytical grade.

### 2.2. Preparation of CNFs

CNFs were prepared using a mechanical grinding method according to the process described by Nie et al. [27]. Initially, the bleached bagasse pulp board was soaked in deionized water overnight at room temperature and was then disintegrated by a fiber disintegrator (AG 04, Estanit GmbH, Muhlheim, Germany) for 30 min to obtain disintegrated pulp with 1% (*w*/*w*) concentration. The water in the disintegrated pulp was dehydrated and the disintegrated pulp was placed in a refrigerator at 4 °C overnight to balance the moisture levels. Then, the disintegrated pulp with a solid content of 2% (*w*/*w*) was ground using an ultrafine grinder (MKZA10-15J, Masuko Sangyo, kawaguchi, Japan) with -100-µm disc spacing at a speed of 1500 rpm to obtain MFC suspensions. After 10 grinding cycles, a CNFs suspension with a solid content of 2.56% (*w*/*w*) was obtained and stored in a 4 °C refrigerator for further use.

### 2.3. Characterization of CNFs

The morphology of CNFs was observed using a transmission electron microscope (TEM). The CNFs suspensions were diluted to concentrations of 0.008% (*w*/*w*) with deionized water and were ultrasonically dispersed for 30 min. A drop of the dispersed CNFs suspension was deposited on a carbon-coated grid and was then stained with 1.5% (*w*/*w*) phosphotungstic acid water for 15 min in a dark place. The dried grid was observed by a TEM (HT7700, Hitachi, Tokyo, Japan) with an acceleration voltage of 100 kV.

### 2.4. Preparation Chitosan/OEO Films Enhanced by CNFs

Chitosan (2%, *w*/*v*) was dispersed in a glacial acetic acid solution (1%, *v*/*v*) and magnetically stirred at 250 rpm for 8 h at room temperature to completely dissolve the chitosan. The pure OEO with addition amounts of 0%, 1%, 2%, 3% (*w*/*w*, chitosan-based) and Tween-80 (40% *w*/*w*, OEO-based) were added to the chitosan solution and stirred by a high-shear homogenizer (Unidrive-Model×1000D, CAT M.Zipperer GmbH, Ballrechten-Dottingen, Germany) at 8000 rpm for 10 min to obtain film-forming solutions. Tween-80 was used as surfactant to facilitate emulsion formation and stability. After ultrasonic deaeration for 1 h, 30-g film-forming solutions were cast onto Teflon plates (150 mm × 150 mm) and dried in an oven at 35 °C for 2 days. All dried film samples were removed from the molds and were stored at 25 °C and 50% RH until the antibacterial activity was evaluated.

To evaluate the enhancement of the CNFs on the properties of the chitosan/OEO biocomposite film with the best antimicrobial activity, the optimal added OEO amount was first determined. Two percent (*w*/*w*, chitosan-based) OEO was added in the follow-up experiments, based on the results (Section 3.2). The CNFs with a concentration of 2.56 wt % were dispersed in distilled water by a high-shear homogenizer at 13,000 rpm for 6 min to obtain a CNFs suspension with a concentration of 1.0 wt %. The 0%, 20%, 40%, 60% CNFs (*w*/*w*, chitosan-based) were added to the chitosan solution and stirred by a high-shear homogenizer at high speed (13,000 rpm) for 6 min to obtain mixtures of chitosan and CNFs. In addition, the 2% OEO (*w*/*w*, chitosan- and CNFs-based) and Tween-80 (40% *w*/*w*, OEO-based) were added to the mixture and homogenized using a high-shear homogenizer at 8000 rpm for 10-min to obtain film-forming solutions which contained CNFs. The film-forming solutions were deaerated, cast and dried as described above to obtain the chitosan/OEO/0% CNFs (COC_0_), chitosan/OEO/20% CNFs (COC_20_), chitosan/OEO/40% CNFs (COC_40_) and chitosan/OEO/60% CNFs (COC_60_) films.

### 2.5. Antimicrobial Properties

Antimicrobial activities of the sample films were evaluated using growth inhibition rates and disk inhibition zone assays. The *Escherichia coli* and *Listeria monocytogenes* cultures were regenerated through the exponential growth phase (24 h) in nutrient broth and brain heart infusion (BHI) broth in incubators at 37 °C and 75% RH to obtain a bacterial suspension with a concentration of 10^8^ CFU/mL.

Before the inhibition rate test, the bacterial suspension was diluted to 10^5^ CFU/mL by phosphate buffer saline (PBS) and the sample film was chopped into fragments and placed under ultraviolet sterilization for 1 h. Then, 100 mg of sample film fragments were placed into 5 mL of an *E. coli* and *L. monocytogenes* suspension of 10^5^ CFU/mL and then shaken at 250 rpm in a water bath shaker at 37 °C for 2 h. After 2 h of contact time, 0.1 mL of *E. coli* and *L. monocytogenes* suspension diluted to 10^2^ CFU/mL (with the sample film) was uniformly coil-coated on the nutrient surfaces and BHI agar plates, respectively. The plates were incubated for 24 h at 37 °C and 75% RH. The inhibition rates of *E. coli* and *L. monocytogenes* growth were calculated by the following equation:

Growth inhibition rate (%) = (A − B)/A × 100%(1)

where A and B are the bacterial counts from the control and the sample films, respectively. All values were averaged from three parallel experiments.

The bacterial suspension was diluted by 100 times with PBS to obtain an inoculum which contained approximately 10^6^ CFU/mL for disk inhibition zone assays. All sample films were cut into circular discs of 10-mm diameter. All culture media were double-layered in which the concentration of the upper agar was 0.5 times that of the underlying agar (BHI agar was used as the medium for *L. monocytogenes* and nutrient agar as the medium for *E. coli*). One hundred microliters bacterial cultures with 10^5^ CFU/mL were uniformly coil-coated on the surface of the BHI and nutrient agar plates and the film discs were placed on plates. The plates were incubated for 24 h at 37 °C and 75% RH. The diameters of the inhibition zones were measured with a vernier caliper.

### 2.6. SEM Analysis

The cross-sectional morphologies of the sample films were observed with a scanning electron microscope (F16502, Phenom, Eindhoven, Netherlands) at 5 kV. The cross-sections of the sample films were exposed by fracturing the films in liquid nitrogen and sprayed with a thin layer of gold under vacuum.

### 2.7. FTIR Spectrum

The chemical structures of sample films were characterized using a Fourier-transform infrared spectrometer (TENSEOR 27, Bruker, Ettlingen, Germany) over a range of 400–4000 cm^−1^ that was operating in attenuated total reflection (ATR) mode and with a resolution of 4 cm^−1^.

### 2.8. X-ray Diffraction (XRD)

X-ray diffraction (XRD) spectra of the sample films were measured by an X-ray diffractometer (MiniFlex600, Rigaku Corporation, Tokyo, Japan) with Cu Kα radiation (λ = 0.15418 nm) that was generated at 40 kV and 30 mA. The diffraction patterns of the films were recorded over an angular range of 2θ = 5°–50° at a constant rate of 5°/min.

### 2.9. Thermal Stability

The thermal stability of sample films was measured using a thermal gravimetric (TG) analyzer (STA449F5, NETZSCH, Bayern, Germany) in a nitrogen atmosphere and the films were heated from 30 to 600 °C at a heating rate of 10 °C/min and nitrogen flow rate of 20 mL/min.

### 2.10. Mechanical Property

Sample film thicknesses were measured using a digital micrometer (model 11,248–001, TMI, New Castle, DE, USA). Elongations at the breaking and tensile strengths of the sample films were determined by an electronic universal material testing machine (MODEL 3367, Instron, MA, USA). The films were cut into strips (100 mm × 15 mm). Stretching rates and initial grip separations were set to 10 mm/min and 50 mm, respectively.

### 2.11. Light Transmittance

The light transmittance of the developed biocomposite films was measured using a UV-visible spectrophotometer (Specord 50 Plus, Analytik Jena, Jena, Germany) in a wavelength range from 380 to 800 nm. An empty quartz cuvette was used as the blank. Each film sample was cut to 9 mm × 40 mm and was attached to the wall of the cuvette before measurement.

### 2.12. Barrier Properties

The oxygen transmission rate (OTR) values of the developed biocomposite films were measured by an automated oxygen permeability testing instrument (OX-TRAN 2/21, MOCON, Inc., Minneapolis, MN, USA) with a coulometric oxygen sensor method and followed the ASTM D3985 standard [28]. OTR is the volume of permeant oxygen passing through a film per unit surface area and time under equilibrium with testing conditions, and the unit of OTR was expressed as cc/m^2^·day [29]. The test area of the samples was 5 cm^2^ and the tests were performed at 23 °C and 50% RH. The test gas was oxygen with a flow rate of 20 mL/min while a mixture of nitrogen (98%) and hydrogen (2%) was used as the carrier gas with a flow rate of 10 mL/min. The test mode was convergence by cycles.

The water vapor transmission rate (WVTR) of the developed biocomposite films was measured using a water vapor permeability testing instrument (TSY-T1, Labthink, Jinan, China). WVTR is the weight of permeant moisture passing through a film per unit surface area and time under equilibrium with testing conditions, and the unit of WVTR was expressed as g/m^2^·day [29].The film was cut into ∅100 mm circular pieces and was placed onto a permeability cup with a 63.58 cm^2^ testing area. The cup was previously filled with 10 mL of distilled water (RH 100%). The cup was sealed and placed into the dry chamber of the instrument at 38 ± 0.6 °C and 10% RH. The sealed cup was weighed periodically (0.001 g) until testing was complete. Water vapor amounts transported into the dry chamber were determined by the weight loss of the cup.

### 2.13. Statistical Analysis

The data were reported as mean ± standard deviation and analyzed by one-way analysis of variance (ANOVA) and Duncan’s multiple range tests using the SPSS 22.0 statistical package for Windows (IBM SPSS Statistical software, Inc., Chicago, IL, USA). The significance level was always set to *p* < 0.05.

## 3. Results and Discussion

### 3.1. Characteristic of CNFs

**Figure 1:**
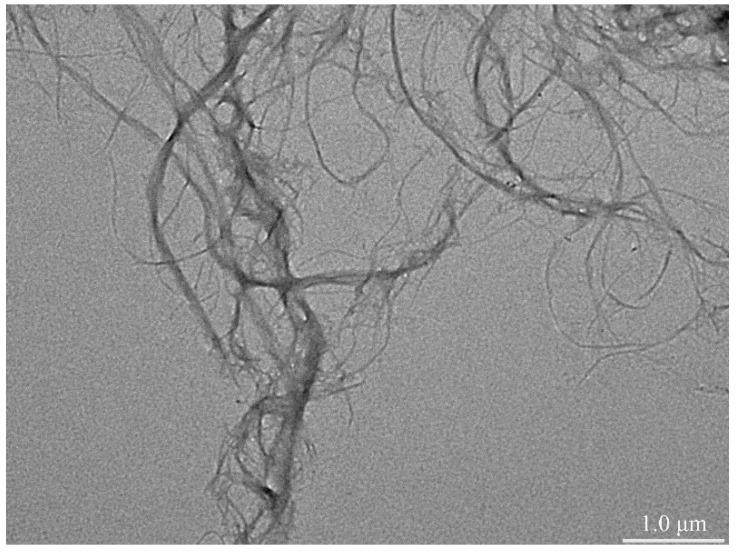
Transmission electron microscopy (TEM) image of cellulose nanofibrils (CNFs).

The morphology of the CNFs is shown in Figure 1. The TEM image shows that the lengths of the prepared CNFs ranged from 200 nm to several microns, diameters ranged from 20 to 50 nm, length–diameter ratios were greater than 50, and there were intertwinements between the long fibrils [30].

### 3.2. Antimicrobial Properties

**Figure 2:**
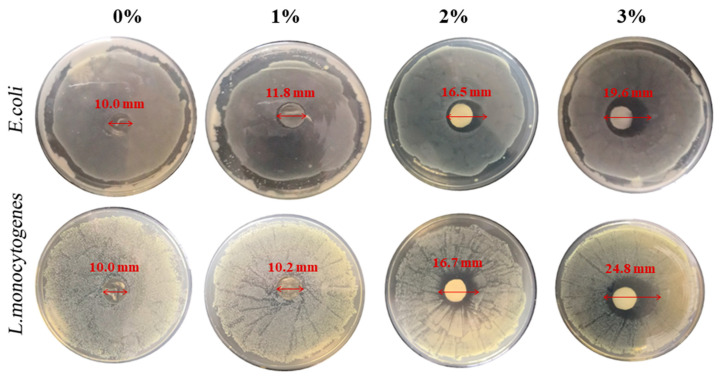
Area of inhibition zone of the films with the different addition amount of oregano essential oil (OEO) against *E. coli* and *L. monocytogenes*.

**Figure 3:**
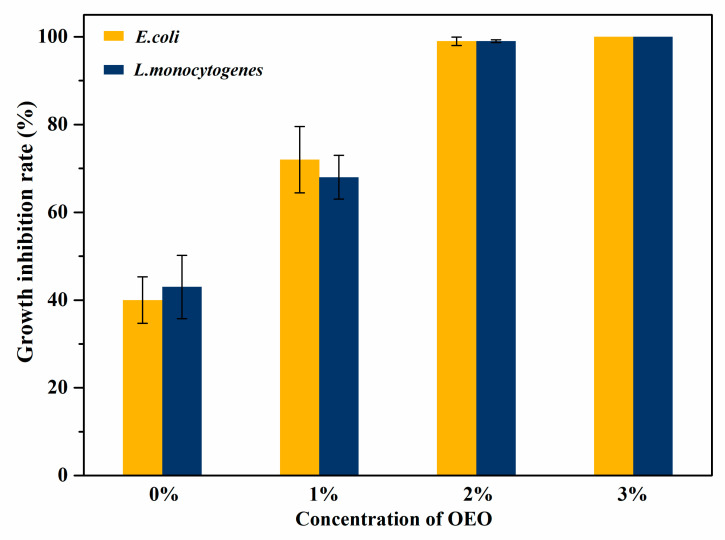
Growth inhibition rate of the films with the different addition amount of OEO against *E. coli* and *L. monocytogenes*.

To determine the optimal addition amount of OEO in the following experiments, 1%, 2% and 3% OEO were added to the chitosan/OEO films. The antimicrobial activities of the chitosan/OEO films with different amounts of OEO against *E. coli* (Gram-negative) and *L. monocytogenes* (Gram-negative) were evaluated and the results are shown in Figure 2 and Figure 3. As is shown in Figure 2, there was no clear inhibition zone either on *E. coli* or on *L. monocytogenes* around the pure chitosan film (0% OEO). With increased OEO addition amounts, the areas of the inhibition zones against both *E. coli* and *L. monocytogenes* gradually increased. The antibacterial properties of the chitosan/OEO films significantly improved by adding OEO and demonstrated that OEO had good inhibition properties against *E. coli* and *L. monocytogenes*. As shown in Figure 3, it is worth noting that the inhibition rates against *E. coli* and *L. monocytogenes* of pure chitosan film (0% OEO) reached 40% and 43%, respectively. This suggested that pure chitosan film exhibited certain antibacterial properties toward *E. coli* and *L. monocytogenes,* but that the antibacterial properties were not obvious. However, the poor antibacterial properties of pure chitosan film cannot meet the requirements for packaging materials for some perishable foods. When the addition amounts of OEO were 2% and 3%, the growth inhibition rates of the developed films against *E. coli* and *L. monocytogenes* reached 99%. However, essential oils usually have a strong pungent, are volatile and excessive levels of essential oils in food packaging as antibacterial agents may affect the original food flavor [31]. Therefore, the optimal addition amount of OEO was chosen to be 2% to ensure a high antibacterial rate. The excellent antibacterial properties indicated that chitosan/OEO biocomposite films have the potential to be used as antimicrobial packaging materials to extend food shelf life.

### 3.3. SEM Analysis

**Figure 4:**
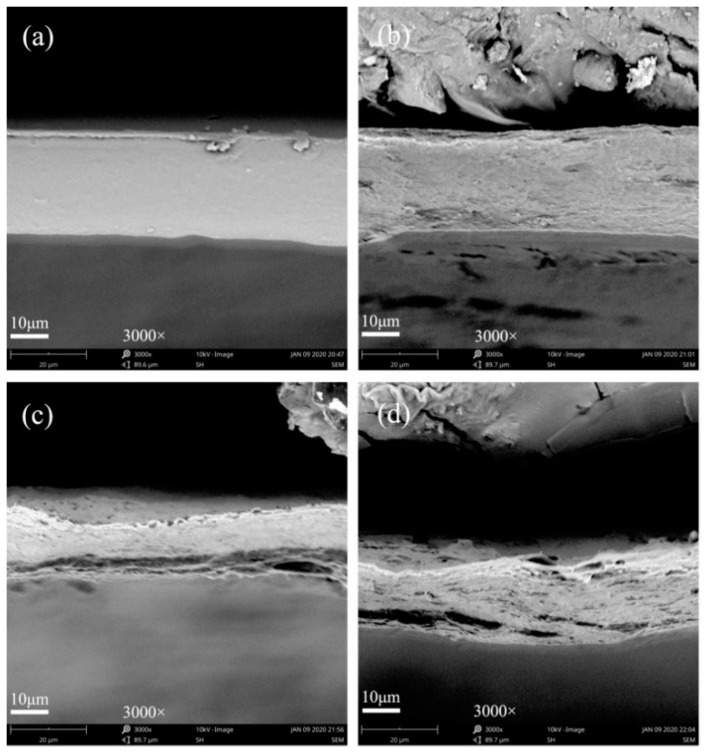
SEM images of the cross-section of the (**a**) chitosan/OEO/0% CNFs (COC_0_) film; (**b**) chitosan/OEO/20% CNFs (COC_20_) film; (**c**) chitosan/OEO/40% CNFs (COC_40_) film and (**d**) chitosan/OEO/60% CNFs (COC_60_) film.

SEM was used to observe the microstructures of the developed chitosan/OEO/CNFs films to analyze the influence of adding CNFs on the film morphologies. Figure 4 shows the cross-sectional morphologies of the COC_0_, COC_20_, COC_40_ and COC_60_ films. The COC_0_ film showed a tight and homogenous structure and few pores may be caused by the volatilization of the OEO [32]. The fibrous-network structure of the films became more obvious with increased addition amounts of CNFs. This behavior was due to the hydroxyl group on the CNFs chains through hydrogen bonding interactions with chitosan. Moreover, the fibers overlapped each other and formed a dense three-dimensional network structure.

### 3.4. FTIR Spectrum

**Figure 5:**
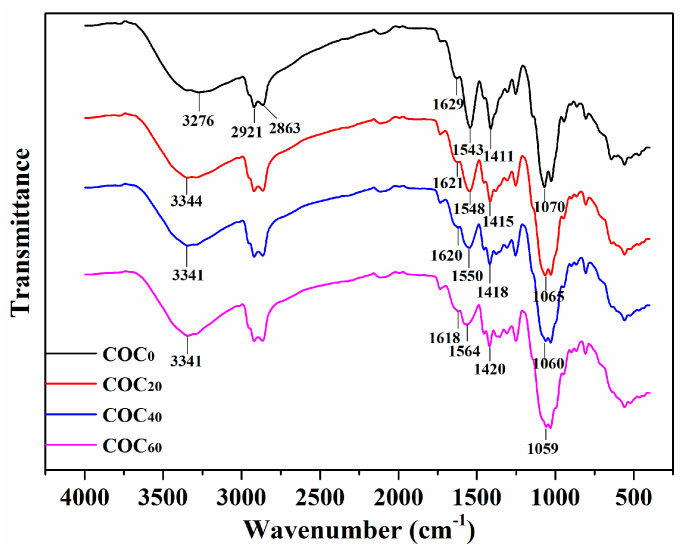
FTIR spectrum of the COC_0_ film, COC_20_ film, COC_40_ film and COC_60_ film; (COC_0_: chitosan/OEO/0% CNFs; COC_20_: chitosan/OEO/20% CNFs; COC_40_: chitosan/OEO/40% CNFs; COC_60_: chitosan/OEO/60% CNFs).

FTIR spectra are widely used to analyze changes in chemical structure and components of co-composites. The FTIR spectra of all sample films with different addition amounts of CNFs are shown in Figure 5. The characteristic peaks at 1629, 1543 and 1411 cm^−1^ were assigned to C=O stretching (amide I), N–H bending (amide II) and C–N stretching (amide III), respectively [33]. These are the characteristic peaks of chitosan which appeared in all spectra of all films and confirmed that chitosan was the matrix material for all films. In the spectra of the COC_0_ films, the broad peak at 3276 cm^−1^ was attributed to O–H and N–H stretching of chitosan. After the CNFs were incorporated with the chitosan, the position of the broad peak of the COC_20_, COC_40_ and COC_60_ films was shifted to around 3341 cm^−1^ which was due to overlapping of the O–H bonds in both CNFs and chitosan. The peak at 1070 cm^−1^ was related to the C–O–C stretching vibration of chitosan in the COC_0_ film spectra [34]. However, the C–O–C stretching vibration of the films containing CNFs was shifted to around 1059 cm^−1^ which was due to the overlap of the C–O–C bonds in both CNFs and chitosan [35]. These results demonstrated that there are strong hydrogen bonds between CNFs and chitosan in the molecular chain. The peaks in the region of 2921 and 2863 cm^−1^ were attributed to symmetric and asymmetric methylene stretching vibrations, respectively. Wu et al. [36] also found that the peaks at 2928 and 2864 cm^−1^ became stronger in a gelatine–chitosan film with 4% OEO. These results indicated that OEO was successfully introduced into the films.

### 3.5. X-ray Diffraction (XRD)

**Figure 6:**
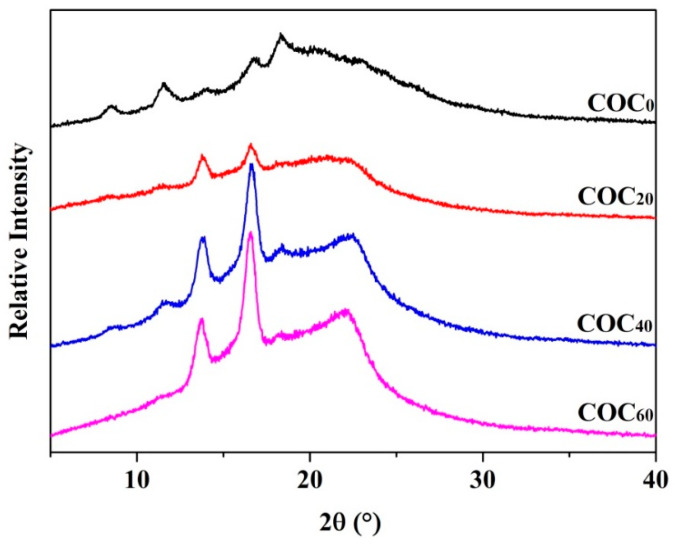
X-ray diffraction patterns of the COC_0_ film, COC_20_ film, COC_40_ film and COC_60_ film; (COC_0_: chitosan/OEO/0% CNFs; COC_20_: chitosan/OEO/20% CNFs; COC_40_: chitosan/OEO/40% CNFs; COC_60_: chitosan/OEO/60% CNFs).

The XRD patterns of the chitosan/OEO/CNFs films with different added amounts of CNFs are shown in Figure 6. Soni et al. [37] reported that the characteristic peaks for pure chitosan films were near 2θ = 9.77° and 19.88°. However, the positions of these characteristic peaks of chitosan-based films with OEO were slightly shifted (e.g., 2θ = 8.48° and 18.32°) which indicated that the original crystalline structure of the chitosan was destroyed [38]. After the addition of CNFs, the characteristic peaks of the CNFs appeared in the region of 2θ = 16.5° and 22°. The strengths of these peaks and the crystallinity of the films slightly increased with increased CNFs content and could be due to the ordered accumulation of chitosan chains on the surface of the crystalline domains of the CNFs [39]. Fernandes et al. [39] also reported that increased bacterial cellulose contents promoted crystallization of chitosan chains as observed in the diffractograms of water soluble chitosan/bacterial cellulose nanocomposite films. The strength of the crystalline peak increased with increased CNFs content which was due to the high biocompatibility between chitosan and cellulose [40].

### 3.6. Thermal Stability

**Figure 7:**
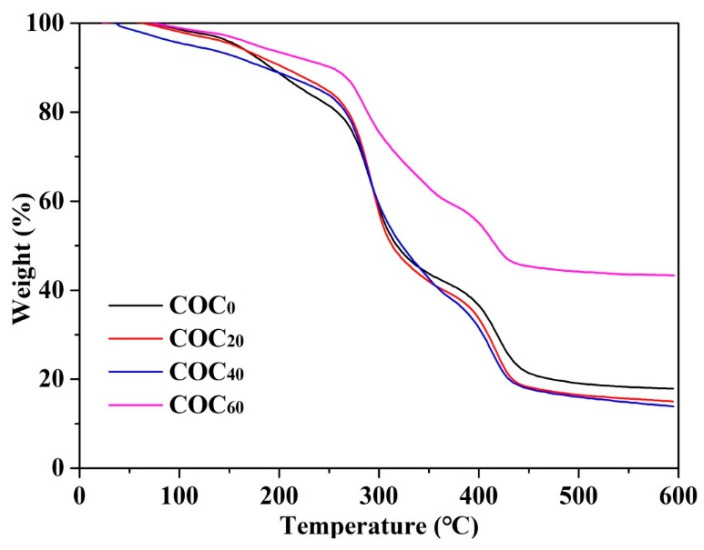
Thermal gravimetric (TG) curve of the COC_0_ film, COC_20_ film, COC_40_ film and COC_60_ film; (COC_0_: chitosan/OEO/0% CNFs; COC_20_: chitosan/OEO/20% CNFs; COC_40_: chitosan/OEO/40% CNFs; COC_60_: chitosan/OEO/60% CNFs).

The thermal stabilities of the sample films were examined by TG to evaluate the effect of CNFs addition on the thermal degradation behavior of the films. Figure 7 shows the TG curves of the COC_0_, COC_20_, COC_40_ and COC_60_ films. The TG curves of all sample films showed the first stage of weight loss occurred between 90 and 250 °C which was associated with water evaporation. Similarly, the weight loss of cassara starch/chitosan/gallic acid films reinforced by CNFs occurred in the range of 90–225 °C and was also due to the weight loss of the absorbed moisture in the films [41]. The second degradation stage consisted of the disaggregation of chitosan molecules or/and disaggregation of cellulose chains which occurred between 250–340 °C. The third stage, between 340 and 500 °C, was due to oxidation of the char or/and breakdown of glucose units in CNFs. All sample films showed similar thermal behavior between 250 and 500 °C and therefore, the effect of CNFs on the thermal stability of chitosan/OEO/CNFs biocomposite films was insignificant [3]. However, the total residues of the COC_60_ films at 600 °C were the highest which indicated that 60% addition of CNFs reduced the rate of char oxidation and shifted the char oxidation to higher temperatures [42]. Therefore, the COC_60_ films are more suitable for food packaging applications even when used at a relatively high temperature.

### 3.7. Mechanical Property

Tensile strength (TS) and elongation at break (EB) are fundamental properties for food-packaging films to resist the stresses and strains that the material may endure during food storage and transportation. Xu et al. [43] shown that the carboxylated CNF significantly enhanced the tensile strength of plasticized hemicelluloses/chitosan-based edible films. The mechanical properties of the COC_0_, COC_20_, COC_40_ and COC_60_ films are shown in Table 1. The TS of the COC_0_ film was determined to be 7.71 MPa and the TS of the COC_20_, COC_40_ and COC_60_ films increased to 10.24, 13.79 and 16.80 MPa, respectively (*p* < 0.05). The high TS of the films containing CNFs may result from the large aspect ratio of CNFs [43] and the stronger interfacial interaction between the chitosan and chains of CNFs [22]. Moreover, the result was also related to the mentioned in Section 3.5, the strength of crystalline peak increased in the chitosan amorphous matrix after addition of CNFs [39]. This may be because the intermolecular hydrogen bonding of chitosan was replaced by the new, strong hydrogen bonding between the hydroxyl groups in the CNFs and the hydroxyl groups in chitosan. Therefore, CNFs can be used as a good filler to enhance the mechanical strength of chitosan films. Khan et al. [3] observed a decrease in the EB values of chitosan films from 8.58% to 6.28% due to the addition of nanocrystal cellulose (NCC). In this work, the EB value was determined to be 31.31% for the COC_0_ film. Compared to the COC_0_ film, the EB was significantly reduced by 5.14%, 5.63% and 4.48% for the COC_20_, COC_40_ and COC_60_ films, respectively (*p* < 0.05). These results are attributed to the strong hydrogen bonding and electrostatic interactions between CNFs and the chitosan matrix [37].

### 3.8. Optical Properties

**Figure 8:**
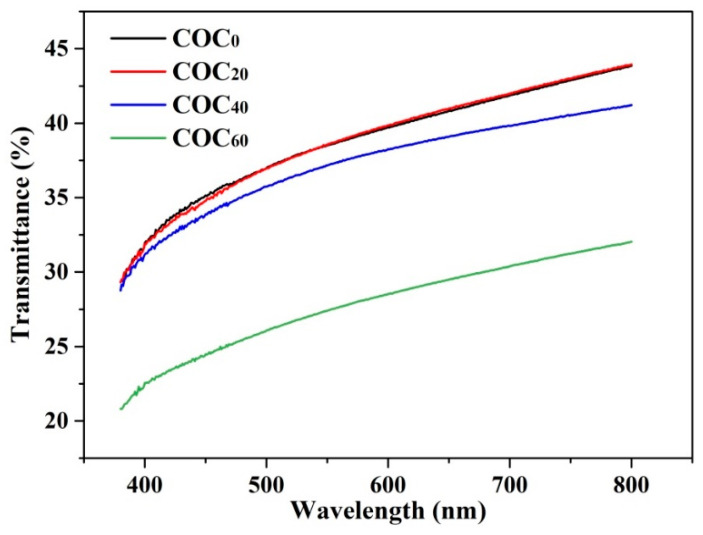
Light transmittance of the COC_0_ film, COC_20_ film, COC_40_ film and COC_60_ film; (COC_0_: chitosan/OEO/0% CNFs; COC_20_: chitosan/OEO/20% CNFs; COC_40_: chitosan/OEO/40% CNFs; COC_60_: chitosan/OEO/60% CNFs).

The transparency of packaging films is important because light can lead to oxidation of nutrients including vitamins, fats and oils and can affect food quality. At the same time, packaging materials also need to have a certain amount of light transmittance to enable consumers to view the packaged products. The light transmittance spectra of the sample films are shown in Figure 8. The light transmittance of the COC_0_ film was the highest of all films which suggested that the light barrier effect of the COC_0_ film was poor. In addition, the light transmittance of the COC_0_ film was 39.73% at 600 nm (center of visible light spectrum) which was lower than most pure chitosan films mentioned in other research [34]; this indicated that the presence of OEO reduced the transmittance of the films [43]. With increasing CNFs contents, the light transmittance of the films decreased, and the opacity increased. This indicated that the addition of CNFs decreased the transparency of the films. The addition of 60% CNFs improved the light barrier effect the COC_60_ film which had the lowest light transmittance (28.53%) at 600 nm. These results suggested that CNFs were densely packed in the chitosan matrix and with compact lap between the fibers, light scattering was prevented by the small interstices between the fibers [44]. All of the results implied that the COC_60_ film has good prospects for food packaging because it has excellent shading properties.

### 3.9. Barrier Properties

**Figure 9:**
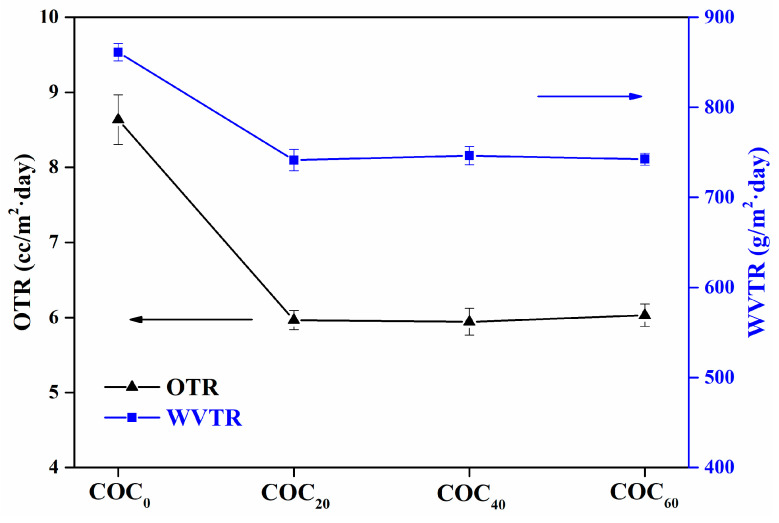
Oxygen transmission rate and water vapor transmission rate of the COC_0_ film, COC_20_ film, COC_40_ film and COC_60_ film; (COC_0_: chitosan/OEO/0% CNFs; COC_20_: chitosan/OEO/20% CNFs; COC_40_: chitosan/OEO/40% CNFs; COC_60_: chitosan/OEO/60% CNFs).

Oxygen and water vapor are the important environmental factors that cause spoilage and deterioration of food during storage. Hence, there is concern about the barrier properties of oxygen and water vapor in food packaging materials. Figure 9 shows the barrier properties of the COC_0_, COC_20_, COC_40_ and COC_60_ films. As shown in Figure 9, the WVTR of the COC_20_, COC_40_ and COC_60_ films significant (*p* < 0.05) decreased when compared to the COC_0_ films (861.26 g/m^2^·day). The reduction in WVTR was due to the physicochemical interactions between CNFs and chitosan which led to reduced numbers of hydrophilic groups (–OH) [8]. As mentioned in Section 3.3, there was good biocompatibility between CNFs and the chitosan matrix and a three-dimensional network structure formed between the fibers by producing winding paths for the water vapor molecules and thus led to reduction of WVTR [22].

If the OTR value is in the region of 1–10 cc/m^2^·day, the packaging material is considered to have good oxygen barrier performance [45]. All sample films in this study had OTR < 10 cc/m^2^·day. However, the OTR of the COC_20_ films was 5.97 cc/m^2^·day which were lower than that of the COC_0_ film (8.64 cc/m^2^·day). Their decreased oxygen permeability could be due to the presence of a more tortuous path between the fibers for penetration by oxygen molecules [46]. Compared with the OTRs of the COC_40_ films (5.94 cc/m^2^·day) and COC_60_ films (6.03 cc/m^2^·day) there were comparable. Oxygen permeability was related to the addition of CNFs and was not affected by the CNFs content. Overall, the addition of CNFs to chitosan films shows good oxygen barrier performance and thus indicates that these composite films can be used as barrier packaging for food.

## 4. Conclusions

In this study, a novel biocomposite packaging film with good antibacterial activities in addition to good mechanical and barrier properties was successfully developed based on chitosan as the film matrix, CNFs as a reinforcing filler and OEO as an antibacterial agent. The chitosan film, which contained 2% OEO, exhibited significant antimicrobial activity and its growth inhibition rates against *L. monocytogenes* and *E. coli* reached 99%. The fibers overlapped with each other in the chitosan matrix to form a dense three-dimensional network structure that was observed by SEM. The FTIR spectrum showed that there were strong hydrogen bonds between CNFs and chitosan in the molecular chain. The TS of the chitosan/OEO film increased with the addition of CNFs. CNFs improved the barrier performance of the chitosan/OEO film to light, oxygen and water vapor by reducing light transmittance, oxygen permeability and water vapor permeability. However, the effect of CNFs on the thermal stability of the chitosan/OEO film was insignificant. The developed chitosan/oregano essential oil/CNFs biocomposite film can be used as an antibacterial and barrier materials in the field of food packaging. It has the potential to improve food quality and extend food shelf life.

## Figures and Tables

**Table 1:** Mechanical property of the COC_0_ film, COC_20_ film, COC_40_ film and COC_60_ film; (COC_0_: chitosan/OEO/0% CNFs; COC_20_: chitosan/OEO/20% CNFs; COC_40_: chitosan/OEO/40% CNFs; COC_60_: chitosan/OEO/60% CNFs).

Film	Thickness (μm)	TS (MPa)	EB (%)
COC_0_	58.80 ± 10.13	7.71 ± 0.62	31.31 ± 1.52
COC_20_	58.60 ± 8.73	10.24 ± 0.44	5.14 ± 0.86
COC_40_	57.60 ± 3.71	13.79 ± 0.29	5.63 ± 0.73
COC_60_	57.40 ± 4.88	16.80 ± 0.66	4.48 ± 0.80
